# The possibility of the emergence of Crimean-Congo virus cases during Eid ul Adha: A troubling situation during a blessed festival

**DOI:** 10.1016/j.amsu.2022.104379

**Published:** 2022-08-20

**Authors:** Abdul Moiz Sahito, Syeda Lamiya Mir, Maria Waseem, Malik Ali Ehtsham Awan, Somina Shaikh, Mohammad Yasir Essar

**Affiliations:** Dow University of Health Sciences, Karachi, Pakistan; Dow University of Health Sciences, Karachi, Pakistan; Dow University of Health Sciences, Karachi, Pakistan; Dow University of Health Sciences, Karachi, Pakistan; Dow University of Health Sciences, Karachi, Pakistan; Kabul University of Medical Sciences, Kabul, Afghanistan

## 1 Introduction

An acute, serious, vector-borne viral hemorrhagic fever is caused by the Crimean-Congo Hemorrhagic Fever (CCHF) virus [1]. The CCHFV virus (genus Nairovirus, family Bunyaviridae) is an enveloped monostranded negative sense RNA virus with a triple segmented genome made up of small, intermediate, and massive RNA segments that, in turn, encode for the RNA-dependent RNA polymerase, the glycoproteins Gn and Gc, and the nucleocapsid protein (N). CCHFV is distinguished by a high degree of genetic variety and intricate evolutionary patterns [2]. Crimean hemorrhagic fever was the term given to the illness when it was first identified in the Crimea in 1944. The disease's current name came about because it was later identified as the illness's primary cause is Congo virus in 1969 [3]. The majority of human infections are spread by the bite of a vector tick, close contact with raw meat, or animal blood. Ixodid ticks, mostly from the Hyalomma genus, are the principal vectors for spreading the virus to humans and animals, with H. marginatum being the most important species in Europe [4]. Direct contact with the blood and other bodily fluids of viraemic individuals and animals is another method of transmission to humans [5]. As a result, those who work in agricultural or veterinary industries and come into touch with fresh animal meat and blood like slaughterhouse workers are at more risk of contracting an illness. During CCHF epidemics, healthcare workers are regarded as a high-risk category for morbidity and mortality [6,7].

The most typical signs and symptoms are a sudden onset of fever, chills, trembling, myalgia, headaches, nausea and vomiting, stomach pain, and arthralgia [3].

Asia, the Middle East, the Mediterranean, Africa, and some regions of south-eastern Europe have all recorded CCHF [8].

In Pakistan, the very first case of CCHF was recorded in 1976, and 14 subsequent cases were documented between 1976 and 2010 [9]. Since then, there has been a significant rise in the occurrence of CCHF [10]. Between January 2014 and May 2020, the National Institute of Health, Islamabad, Pakistan, confirmed 356 CCHF cases across the country, with a 25% mortality rate [11]. 38% of these cases were reported from Balochistan, followed by 23% from Punjab, 19% from Khyber Pakhtunkhwa, 14% from Sindh, and 6% from Islamabad [11]. Zohaib et al. found 2.7% of CCHF seroprevalence in Pakistan, with a higher frequency in rural people, presumably due to increased contact to animals [12]. A total of four confirmed cases of CCHF have been recorded up to the end of June 2022. (02 cases each from Sindh and Punjab) [13].

This article aims to provide a commentary that aware people of the alarming situation of the CCHF's spread across the countries and the possible measures that can be taken to prevent the upcoming challenges.

## 2 Prevalence of CCHF in Pakistan

CCHF is a tick-borne viral illness with a wide geographical dissemination [8]. The CCHF virus, which is thought to cause a new illness CCHF, is the second most extensively spread virus among hemorrhagic fever viruses, behind the dengue virus [8]. In endemic areas, CCHF is connected with the Muslim holy feast of Eid-ul Azha. Before Eid-ul- Azha, a significant number of animals are transported from various parts of Pakistan for sacrifice. Increased interaction between people who are not normally involved in animal husbandry or meat handling raises the risk of CCHF [8]. According to a WHO study from 2017, Pakistan is a risk region, with 5–49 cases of CCHF documented per year [8]. After Turkey, Russia, and Iran, Pakistan is considered an endemic nation for CCHF, having the fourth highest recorded incidence of CCHF infection in Asia [8].

## 3 Challenges due to co-existing COVID-19 burden

As the preventive measures and management plans for CCHF are taken into consideration, the co-existing burden of COVID-19 and its consequences on the current situation can't be overlooked. The global efforts that have been undertaken for effective dealing with the pandemic such as social distancing, public lockdowns, transportation shutdowns and healthcare regulations have given rise to a number of negative effects that remain a hindrance in the management of public health distresses other than COVID-19 such as restricted access to healthcare, exhausted public health surveillance capacities, ineffective distribution of healthcare resources leaving little room for accommodation of new management and response plans. According to a report, utilization of healthcare resources for conditions not associated with COVID-19 has been remarkably decreased globally [14]. Together with this, international funding, and delivery of healthcare facilities to areas in need have been considerably compromised due to global disruption of communication, making the formulation and implementation of new healthcare strategies difficult. Pakistan currently has a double COVID-19 burden, which strains the country's already overburdened healthcare system. As COVID-19 containment is the main focus of all health authorities' initiatives in Pakistan, there is a strong likelihood that other infectious illnesses would be overlooked. It should be underlined that the nation is not immediately ready to handle any new outbreak. The second-largest city in Pakistan and one of the COVID-19 hotspots, Lahore, has recorded almost 20 000 cases of typhoid, placing further strain on the healthcare system. Since Eid ul Adha is thought to be the time when disease transmission is most likely to occur, the emergence of CCHF poses an additional hazard during the pandemic. In the WHO's Eastern Mediterranean Region, Pakistan is rated fourth for CCHF cases, behind Turkey, Iran, and Russia [15].

In addition, detrimental effects of global and household economic downturn due to COVID-19 together with the social fear regarding the spread and contraction of COVID-19 with the consequent hesitation of general population to reach out to healthcare facilities continue to impact the adequacy of healthcare infrastructure negatively, the soundness of which is very important for effective application and fruitfulness of future healthcare strategies.

## 4 Regulations for slaughtering animals during Eid Al Adha by different countries

Certain regulations regarding animal slaughtering practices such as environmental hygiene and prompt management plans of CCHF have been successfully implemented in a number of Muslim countries for adequate prevention and control of disease as discussed below.

• **Kingdom Of Saudi Arabia**

KSA takes extensive measures during Hajj for proper animal slaughter as is indicated by the establishment of a Saudi Government partnership with the WHO Collaborating Centre for Mass Gatherings Medicine, the Global Center for Mass Gathering Medicine, Gulf Co-operation Council states, UK universities, and public health institutions globally named as Global Center for Mass Gathering Medicine [16]. As the season of Hajj ensues, 24 formed committees work in a syncytium for the maintenance of public health [17]. Together with this, a Supreme Hajj committee coordinates the Hajj plan and lays recommendations for developing Hajj facilities while a Hajj Preventive Medicine Committee that manages preventive measures taken to minimize public health risk including strict control of entry portals for all pilgrims [18].

• **Iran**

Since 2000, CCCHF has shown to be prevalent in 23 out of 30 provinces of Iran. Since 2000, we have shown the disease to be prevalent in 23 out of 30 provinces of Iran [19]. For adequate management and control of CCHF during Eid-Al-Adha, the surveillance system protocols demand the slaughtering to be exercised in defined industrial slaughterhouses with appropriate PPE usage. Iran Veterinary Organization coordinates with municipalities of the city for the provision of temporary slaughtering accommodation facilities.

To ensure minimal transmission, the domestic livestock transportation takes place under strict supervision of IVO with adequate assumption of preventive measures such as use of insecticide [20].

• **Turkey**

Government Legislations play quite an important role in application of Eid-Al-Adha related protocols of animal slaughter all over the country. Attainment of food chain identification and a veterinary health certificate is made necessary as legal requirement for the trade of livestock. The Ministry of Food, Agriculture and Livestock manages hygienic slaughtering and handling of meat through spreading public awareness. The legislations make it mandatory to use designated marketplaces for the trade of livestock to ensure minimal exposure to disease causing ectoparasites [20].

## 5 Prevention of CCHF

Avoiding tick bites, employing PPE, and controlling CCHF in animals by utilizing acaricides in cattle production sites are the key preventive methods for CCHF.

• PREVENTING CCHF IN TICKS AND ANIMALS

It is challenging to prevent and control CCHF in tick hosts and other animals, due to the asymptomatic nature of the disease in animals and the prevalence and abundance of ticks in endemic areas. Before slaughtering animals, a 14 day quarantine and acaricides are effective for controlling ticks. But as for now, no vaccines are available to protect animals [21].

• PREVENTING ANIMAL MOVEMENTS

*Trans*-border transmission should be prevented by official laws and charges put in place by local officials. Unregulated livestock movements across endemic nations should be prohibited. This will aid in preventing the international spread of new antigenic variations of the virus [22].

• PREVENTING TICK TO HUMAN TRANSMISSION

All biological stages of ticks consume human blood, with the exception of the egg stage. Rural residents and agriculturists should wear light-colored protective apparel that makes ticks identifiable. You can also use insect repellents, and you should periodically check your skin and clothing for ticks. Fine point tweezers should be used to remove ticks as soon as they attach. The bite site should then be properly cleaned with soap and water, and an antiseptic should be given to the hands and bite region. If a person develops an inexplicable illness with a fever after visiting a tick-infested area, they should consult a doctor [22].

• PREVENTING ANIMAL TO HUMAN TRANSMISSION

Two weeks prior to slaughter, endemic animals may receive pesticide treatment to prevent potential tick infection. Handling an animal's hide without gloves carries a danger of disease transmission that people need to be aware of. Animal excrement and blood shouldn't be dumped in streams and waterways [20].

• PREVENTING HUMAN TO HUMAN TRANSMISSION

When providing treatment to CCHF patients, healthcare workers are at risk for occupational infections; the first such cases were recorded in Pakistan and later from numerous Eurasian nations. In order for HCWs to adopt the essential PPE and infection control procedures, there must be increased awareness of CCHF and early detection of it [23].

## 6 Recommendations

From these resources it can be learned that protective measures during handling of livestock is extremely important though there are no significant disease manifestation in domestic animals, but they are reservoirs for continuous infection of ticks in transmission cycle of the disease. Especially during the occasion of Eid-ul-Adha slaughtering should be done in restricted space specialized for slaughtering with appropriate sterilization methods and workers wearing protective clothing. Animals should be transported under special supervision with minimum contact and protective measures like insecticide sprays to reduce the incidence of CCHF. Government of Pakistan should also make certain laws regarding the trade of animals e.g., making veterinary health certificate mandatory. Government should also partner with international organizations such as WHO and seek help from those organizations specially at the time of Eid-ul-Adha. Further government should also use media for spreading public awareness message across the country. Through these measures risk of infection and burden of CCHF can be reduced significantly hence quality of life will be enhanced. Weeks prior to the festival, informative messages can be delivered to the public in the most comprehensible manner possible through verbal and pictorial media outlets. To establish the causal connection between the environmental, biological, and climatic elements that are CCHF drivers and the therapeutic agents for CCHF, more research is required.

## 7 Conclusion

Although Eid has passed but there are several take home messages through which infection can be prevented in upcoming Eids. The implementation of these policies for efficient surveillance, control, and treatment of CCHF depends on the One Health strategy, which promotes cooperation among veterinary experts, infectious disease doctors, microbiologists, public health experts, and ecologists. It is advised that local authorities limit the amount of human contact with blood and/or fresh meat from sacrificed animals that exposes people to the infection. As it is observed time of Eid-ul-Adha is most vulnerable period of CCHF spread. Many people in Pakistan buy animals several weeks before the Eid and keep them in residential areas eg home which increases the risk of infection. Moreover, many butchers slaughter the animals in houses which increase the contact of people with fluid and fresh meat. At the time of Eid due to high demand of butchers many unprofessional butchers come into play, absence of proper butcher training, improper disposal methods and slaughtering at public places where many people gather around the animal are the factors causing spread CCHF to public. Immediate training programmes for medical personnel, monitoring and reporting animal health, lockdown during Eid al-Adha, public awareness campaigns, advising local authorities on the prompt collection and disposal of waste and carcasses from the slaughtered, ensuring the district-level accessibility of lab equipment and vital medications for the diagnosis and treatment of CCHF, and creating efficient standard operating procedures (SOPs), ensuring slaughtering at designated places such as abattoirs, provisioning of necessary resources to hospitals receiving CCHF patients, encouraging the use of hand sanitizers, soaps and disinfectant sprays during and after slaughtering of animals, and initiating effective vector control programmes are some important measures which need to be taken at the earliest.

## Ethical Approval

N/A.

## Sources of funding

None.

## Author contribution

All authors equally contributed.

## Trial registry number

1. Name of the registry: N/A
2. Unique Identifying number or registration ID: N/A
3. Hyperlink to your specific registration (must be publicly accessible and will be checked): N/A

## Guarantor

N/A.

## Consent

N/A.

## Provenance and peer review

Not commissioned, externally peer reviewed.

## Declaration of competing interest

None declared.
